# A Survey of Location-Allocation of Points of Dispensing During Public Health Emergencies

**DOI:** 10.3389/fpubh.2022.811858

**Published:** 2022-03-10

**Authors:** Nusaybah Alghanmi, Reem Alotaibi, Sultanah Alshammari, Areej Alhothali, Omaimah Bamasag, Kamil Faisal

**Affiliations:** ^1^Department of Information Technology, King Abdulaziz University, Jeddah, Saudi Arabia; ^2^Department of Computer Science, King Abdulaziz University, Jeddah, Saudi Arabia; ^3^Department of Geomatics, King Abdulaziz University, Jeddah, Saudi Arabia

**Keywords:** points of dispensing, location-allocation, public health emergency, healthcare, disaster response and management

## Abstract

Public health emergencies such as disease outbreaks and bioterrorism attacks require immediate response to ensure the safety and well-being of the affected community and prevent the further spread of infection. The standard method to increase the efficiency of mass dispensing during health emergencies is to create emergency points called points of dispensing (PODs). PODs are sites for distributing medical services such as vaccines or drugs to the affected population within a specific time constraint. These PODs need to be sited in optimal locations and have people (demand points) assigned to them simultaneously; this is known as the location-allocation problem. PODs may need to be selected to serve the entire population (full allocation) or different priority or needs groups (partial allocation). Several previous studies have focused on location problems in different application domains, including healthcare. However, some of these studies focused on healthcare facility location problems without specifying location-allocation problems or the exact domain. This study presents a survey of the PODs location-allocation problem during public health emergencies. This survey aims to review and analyse the existing models for PODs location-allocation during public health emergencies based on full and partial demand points allocation. Moreover, it compares existing models based on their key features, strengths, and limitations. The challenges and future research directions for PODs location-allocation models are also discussed. The results of this survey demonstrated a necessity to develop a variety of techniques to analyse, define and meet the demand of particular groups. It also proved essential that models be developed for different countries, including accounting for variations in population size and density. Moreover, the model constraints, such as those relating to time or prioritizing certain groups, need to be considered in the solution. Finally, additional comparative studies are required to clarify which methods or models are adequate based on predefined criteria.

## 1. Introduction

Public health emergencies such as disease outbreaks and bioterror attacks demand an immediate response to save people's lives and to prevent the further spread of infection. The Centers for Disease Control and Prevention (CDC) has created a new approach and organizational unit to meet the challenges in responding to bioterror attacks and other large and complex health emergencies, whether naturally occurring, intended, or casual, such as anthrax attacks (1). Furthermore, being able to respond to a range of future emergencies means that the CDC has to expand its capabilities and responses in terms of scale and speed to cover two global trends: faster and more frequent international travel and the increasing global population (2).

The standard method to increase the efficiency of mass dispensing during health emergencies is to create emergency points, called points of dispensing (PODs) (3). PODs are locations for dispensing medical services, such as vaccines or drugs, to a large number of people, while meeting a specific time constraint (4). PODs are necessary to prevent people who are not sick from becoming infected (5). There are two types of PODs, opened and closed (6). While opened PODs are located at public sites such as schools, closed PODs are operated by a partner organization while the operation continues during the emergency (6).

**Figure 1:**
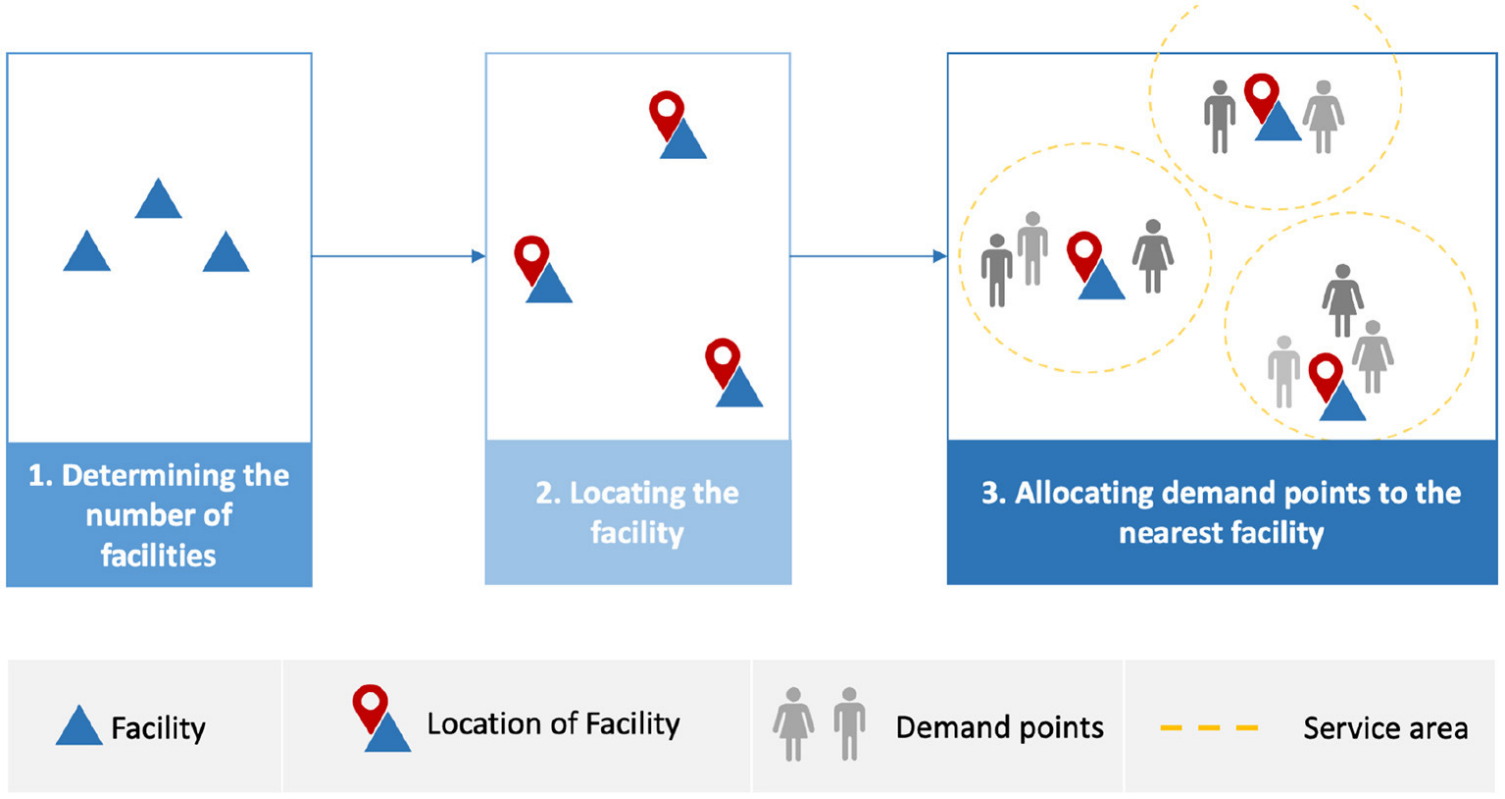
Workflow for location-allocation models.

Poor decision-making while determining the location of healthcare facilities such as PODs results in negative outcomes. These adverse outcomes are not limited to cost and service but also increased infection numbers and deaths due to difficulty in accessing the service (5). A POD need to be sited in a location and, simultaneously, have people assigned to the located facilities; this is known as the location-allocation problem (5). Location-allocation models are used to locate optimal facility locations and allocate demand points to each facility (7). Location-allocation models are operated by planners, who usually determine the number of facilities required (7). The model then locates the facilities before the demand points are allocated to the nearest facility based on a measure such as shortest travel distance or time (7), as shown in Figure 1. Facilities may need to selected to serve the the entire population (full demand points allocation) or populations at high risk, or with different priorities or needs (partial demand points allocation).

### 1.1. Motivation

In March 2020, the World Health Organization (WHO) declared the novel coronavirus disease (COVID-19) a global pandemic (8). During the COVID-19 pandemic, public health authorities required an immediate response plan to access the affected population and ensure they have easy access to the healthcare services and supplies (such as water or food) they need. According to the CDC, distributing food via delivery may be an ideal approach to inhibiting the spread of COVID-19 (9). However, in the case of healthcare services where a physical examination is required, patients need to be able to access physical locations (face to face) to receive services (10).

Given the nature of the fast-spreading of COVID-19, public health authorities across the world needed to respond immediately to ensure the protection of the public, stop the infection from spreading further and provide mass prophylaxis. In the early days of this pandemic, locations needed to be assigned and set up for COVID-19 testing. Then, when COVID-19 vaccines were approved, health facilities and other sites or PODs were needed to provide fair access to the vaccine.

These PODs differ from traditional healthcare facilities, since they involve the provision of easy and fair access for all demand points (5) and have a role in providing medical supplies to protect the public during public health emergencies. However, setting up PODs needs to be done on a large scale and within specific timeframes (4). Thus, locating PODs and allocating the demand points to the designated PODs is a critical and challenging task.

PODs may need to be located to serve the entire population (full allocation) or those who have difficulty accessing PODs and those with different priorities or needs (partial allocation). This survey provides taxonomy-based demand points allocation as full and partial demand points allocation. Which may help improve location-allocation models for efficient PODs assignment while ensuring fair access for all populations or addressing different populations' needs.

### 1.2. Contributions

This survey focuses on location-allocation problems for PODs during public health emergencies. Previous surveys have focused on location problems in different application domains, including the location-allocation of general healthcare facilities (11, 12). Location problems “intend to determine the optimum locations for a set of facilities by minimizing or maximizing some objectives for satisfying the existing and/or projected demand with respect to a set of constraints in some given space (11, 13)”, while location-allocation problems intend to locate facilities and simultaneously allocate demand points to facilities (5). Surveys, such as Ahmadi-Javid et al. (5) and Afshari and Peng (14), have focused on the locations of healthcare facilities without a focus on the location-allocation problems or specific applications such as PODs during public health emergencies. To the best of our knowledge, there exist no studies surveying PODs location-allocation problems during public health emergencies in the form of allocation decisions (full and partial). Thus, the contributions of this paper are as follows:

- Providing a taxonomy for PODs location-allocation problems during public health emergencies based on demand allocation (full and partial);
- Comparing existing solutions based on their key features, strengths, and limitations;
- Discussing the challenges and issues associated with the current solutions for PODs location-allocation during public health emergencies and providing future directions.

The rest of this survey is structured as follows: Section 2 introduces the background of the components of location-allocation problems, location-allocation models and location-allocation solutions. Section 3 presents survey methodology while, Section 4 presents existing solutions to PODs location-allocation during public health emergencies. Section 5 then discusses the challenges of, and potential future research direction for, location-allocation solutions for PODs during public health emergencies. Finally, Section 6 concludes the study.

## 2. Background

### 2.1. Components of Location-Allocation Problems

There are three essential components for location-allocation problems: facilities, locations, and customers (15, 16). Facilities reflect various objects with spatial positions to optimize interaction with preexisting objects; an example of a facility is a school or hospital. Locations are a set of candidate points where facilities can be positioned or located. Finally, ‘customers' is a general term to indicate any objects interacting with facilities. Customers can be users demanding a service from the facility, for example communities requesting a public service in a rural area. The modeling and analysis of interactions between facilities and customers often require the knowledge of their spatial positions (16). In the remainder, we refer to customers as demand points.

### 2.2. Location-Allocation Models

The location problem can be discrete, continuous (5, 17) or network (16, 18). Continuous location problems locate the facility anywhere in a spatial region, while discrete location problems limit facility locations to a set of prespecified candidate points which may include the locations of the demand points (5, 16). Network models implement graph theory to model location problem (16), and for the purpose of composing nodes and links (18). Facilities and demand points can be located only on links or nodes, and any travel between them should be within the network (18).

We focus on the discrete case for several reasons. It is highly flexible in terms of including various economic and geographical features in the models (16). It also provides more natural design results when free land is available and requires locating a new facility at a prespecified place (16). More importantly, using a discrete set of candidate locations is the preferred approach for the majority of location problems and appears frequently in healthcare applications (5). There are two categories of discrete location-allocation problems which are covering-based and median-based problems (5). Table 1 explains the mathematical notations and input parameters used in their mathematical formulations.

**Table 1:** List of mathematical notations and input parameters.

**Variable**	**Description**
*d*_*ij*_	Travel distance or time from demand point *i* to the candidate location *j*.
*h*_*i*_	Demand at point *i*.
*P*	Number of facilities to be located.
*f*_*j*_	Fixed cost to locate facilities at candidate locations *j*.
*v*	Transport costs per unit of demand per distance unit.

#### 2.2.1. Covering-Based Problems

In covering-based problems, each demand point should be withing a particular distance or time range from the facility that serves them. P-centre location problems (PCLPs) are covering-based problems that aim to minimize the maximum travel distances or times between the selected facilities and the demand points. The decision variables of the PCLPs are presented in Table 2, and the formulation is as follows (5, 18):

Formulation of PCLPs


(1) $$\begin{array}{l} \min W \end{array}$$

subject to


(2) $$\begin{array}{l} \underset{j}{\sum }Y_{ij}=1,\;\forall i \end{array}$$

(3) $$\begin{array}{l} \underset{j}{\sum }X_{j}=P \end{array}$$

(4) $$\begin{array}{l} \underset{j}{\sum }d_{ij}Y_{ij}\leq W,\;\forall i \end{array}$$

(5) $$\begin{array}{l} Y_{ij}\leq X_{j},\;\forall i,j \end{array}$$

(6) $$\begin{array}{l} Y_{ij}\in \left\{0,1\right\},\;\forall i,j \end{array}$$

(7) $$\begin{array}{l} X_{j}\in \left\{0,1\right\},\;\forall j \end{array}$$

(8) $$\begin{array}{l} W\geq 0. \end{array}$$

**Table 2:** List of decision variables for PCLPs.

**Variable**	**Description**
*W*	Maximum travel distance from any demand point to its assigned location.
*X*_*j*_	1 if a facility is located at candidate location *j*, otherwise 0.
*Y*_*ij*_	1 demand point *i* is assigned to facility at the candidate location *j*, otherwise 0.

In the formulations listed above, (1) illustrates the first objective of PCLPs, namely, to minimize the maximum travel time or distance between the demand points and the closest facilities. The constraints are listed from (2) to (8), where (2) requires assigning each demand point to only one facility. Constraint (3) limits the number of established facilities. Constraint (4) states the maximum travel time or distance, which means that *W* must be greater than the distance between any demand point and its serving facility. Constraint (5) is the facility that should be opened to serve demand points. Finally, constraints (6), (7) and (8) are the domain constraints. Note that (18) considers *W* a decision variable, while (5) consider it an auxiliary variable.

#### 2.2.2. Median-Based Problems

In this set of location-allocation problems, facilities are located at the candidate locations to minimize the weighted average distance cost among each demand point and the facility to which they are assigned. Median-based problems are divided into p-median location problems (PMLPs) and fixed-charge facility location problems (FCLPs) (5). PMLP aims to locate the facilities while minimizing the weighted travel time or distance. The decision variables of the PMLPs are presented in Table 3, and the formulation is as follows (5, 16, 18):

Formulation of PMLPs


(9) $$\begin{array}{l} \min\underset{i}{\sum }\underset{j}{\sum }h_{i}d_{ij}Y_{ij} \end{array}$$

subject to


(10) $$\begin{array}{l} \underset{j}{\sum }Y_{ij}=1,\;\forall i \end{array}$$

(11) $$\begin{array}{l} \underset{j}{\sum }X_{j}=P \end{array}$$

(12) $$\begin{array}{l} Y_{ij}\leq X_{j},\;\forall i,j \end{array}$$

(13) $$\begin{array}{l} Y_{ij}\in \left\{0,1\right\},\;\forall i,j \end{array}$$

(14) $$\begin{array}{l} X_{j}\in \left\{0,1\right\},\;\forall j \end{array}$$

**Table 3:** List of decision variables for PMLPs.

**Variable**	**Description**
*X*_*j*_	1 if a facility is located at candidate location *j*, otherwise 0.
*Y*_*ij*_	1 demand point *i* is assigned to facility at the candidates locations *j*, otherwise 0.

In the formulations listed above, (9) objective of PMLPs minimizing the total demand-weighted travel time or distance between every demand point and their closest facility. The constraints are listed from (10) to (14), where (10) limits the assignment of each demand point to a single facility. Constraint (11) limits the number of established facilities. Constraint (12) requires a facility to be opened in order to serve demand points. Finally, constraints (13) and (14) are integrality constraints.

FCLPs attempt to minimize total costs in terms of traveling and setting up the facilities. The decision variables of the FCLPs are presented in Table 4, and the formulation is as follows (5, 18).

**Table 4:** List of decision variables for FCLPs.

**Variable**	**Description**
*X*_*j*_	1 if a facility is located at candidate location *j*, otherwise 0.
*Y*_*ij*_	1 demand point *i* is assigned to facility at the candidates locations *j*, otherwise 0.

Formulation of FCLPs


(15) $$\begin{array}{l} \min\underset{j}{\sum }f_{j}X_{j}+v\underset{i}{\sum }\underset{j}{\sum }h_{i}d_{ij}Y_{ij} \end{array}$$

subject to


(16) $$\begin{array}{l} \underset{j}{\sum }Y_{ij}=1,\;\forall i \end{array}$$

(17) $$\begin{array}{l} Y_{ij}\leq X_{j},\;\forall i,j \end{array}$$

(18) $$\begin{array}{l} Y_{ij}\in \left\{0,1\right\},\;\forall i,j \end{array}$$

(19) $$\begin{array}{l} X_{j}\in \left\{0,1\right\},\;\forall j \end{array}$$

In the formulations listed above, (15) illustrates the objective of FCLPs, namely, to minimize the total cost of demand-weighted distance and facility opening. The constraints are listed from (16) to (19), where (16) requires assigning each demand point to a single facility. Constraint (17) requires opening a facility that serve demand points. Finally, constraints (18) and (19) are integrality constraints.

### 2.3. Location-Allocation Solutions

There are three solutions approaches for the location-allocation problems (15): Exact solutions, heuristic methods, and metaheuristic methods. Exact solutions (15, 19, 20), such as branch and bound algorithm, find the optimal solution through systematically examining a large subset of all possible feasible combinatorial set of facility location and demand allocation solutions. On the other hand, heuristic methods (15, 19, 20), such as greedy assignment, find the near-optimal solution by examining only a limited subset of the potential combinations. Finally, metaheuristic methods such as genetic algorithm (GA) (15, 21), provide a general framework and a higher-level procedure to design heuristic algorithms which are more powerful than standard heuristic methods.

## 3. Survey Methodology

This survey focuses on PODs location-allocation during public health emergencies. Thus, two stages were involved in achieving the objectives of the study: examining the results of previous relevant surveys and reviews, and carrying out a literature review. Examining previous surveys helps to understand the topic and identify gaps in survey and review articles. The literature review stage helps find, present and compare research papers proposing a solution for PODs location-allocation during public health emergencies. This stage also analyzes the solution's strengths, limitations and key features to identify particular issues and challenges to guide future directions. This survey focuses on solutions for location-allocation of PODs during public health emergencies; a more detailed explanation and discussion will be presented and provided for these solutions in Section 4.

**Figure 2:**
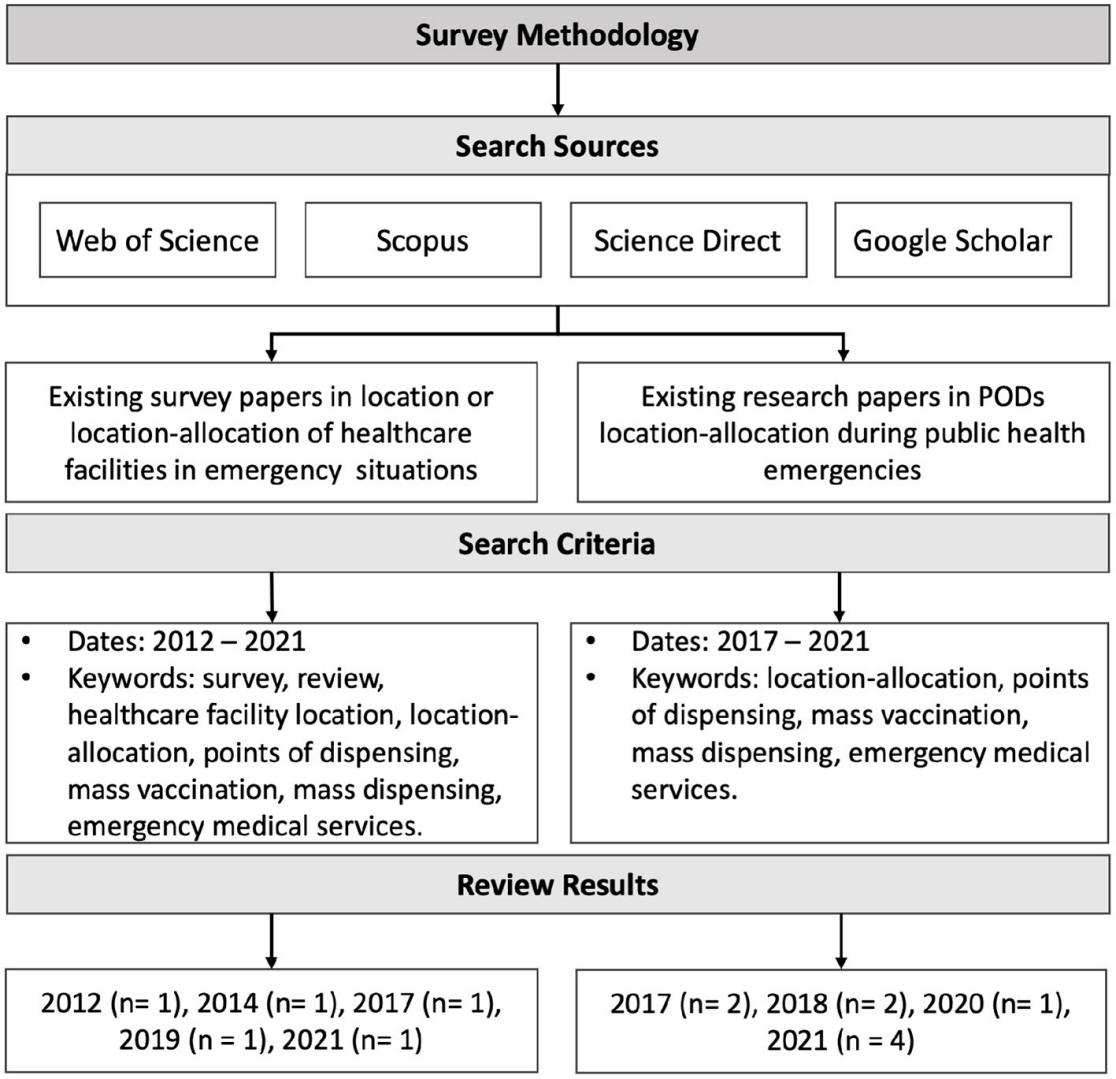
Survey methodology. n refers to number of retrieved papers.

A hybrid search strategy was adopted using systematic and non-systematic approaches to obtain relevant research papers. In non-systematic approaches, the papers were retrieved through backward and forward citations or snowballing. The databases considered in the search were Web of Science, Scopus, and Science Direct. We also search Google Scholar, a web search engine that indexes a full text or metadata of scholarly literature in almost any area. The existing research papers in PODs location-allocation were retrieved using different keywords, namely location-allocation, points of dispensing, mass vaccination, mass dispensing, emergency medical services. All papers had to be in the field of emergency healthcare. While to find existing survey papers about PODs location-allocation, terms such as survey, review and healthcare facility location were used in addition to the previous search keywords. The title and abstract of each paper were evaluated to consider the relevant papers. The search is limited to papers written in English. A detailed overview of the survey methodology is presented in Figure 2.

Most recent and relevant survey and review articles in a range between 2012 and 2021 were examined to obtain an overview of the locations of healthcare facilities or healthcare facility location-allocation in emergency situations. Basar et al. (12) provided a taxonomy for the locations of emergency service stations; the taxonomy covers the type emergency, such as a fire or pandemic disease, as well as use of different objectives, assumptions, constraints, modeling choices, and solutions techniques. Afshari and Peng (14) examined challenges based on the current needs of decision-makers, and methods for locating healthcare facilities to guarantee an optimal solution. They classified literature based on resolved challenges, methods and solutions for facility locations. They also examined the measures used to evaluate the studies in terms of their objective functions and constraints. Ahmadi-Javid et al. (5) presented a paper for both emergency and non-emergency healthcare facility location that included PODs, but presented only three relevant research papers.

Turkoglu and Genevois (11) presented a comparative survey for service facility location problems in different application fields, including healthcare. First, they defined several key features for location problems, such as the number of facilities or objectives. They then defined descriptive dimensions such as application fields, and used these 19 characteristics to present existing solutions. The solutions were categorised based on the application fields and these characteristics were summarized for each field. Moreover, they compared the solutions in different application fields based on these characteristics.

Finally, in the location-allocation problem, Gwalani et al. (22) evaluated and compared four heuristic algorithms to solve the p-median problem in different terms, namely objective function value, time, and stability. Also, they considered the effect of scale (number of sources and destinations) and spatial distribution of destination locations on the performance of these algorithms. Finally, they applied the evaluation in synthetic and real datasets, the latter including resource distribution during bio-emergencies.

## 4. Location-Allocation Models for PODs During Public Health Emergencies

**Figure 3:**
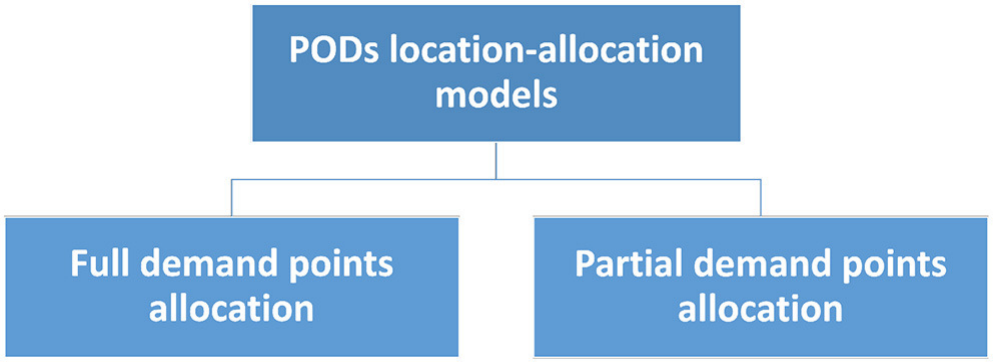
Taxonomy of PODs location-allocation models during public health emergencies.

This section presents the existing research papers for location-allocation models for PODs during public health emergencies. We focused on retrieving the research papers between 2017 and 2021. As a result, there were (2) and (2) research papers from 2017 and 2018, respectively, while in 2020 and 2021, there are (1) and (4) research papers. The existing location-allocation methods for PODs, as shown in Figure 3, are divided based on the allocation of demand points as full demand points allocation and partial demand points allocation.

### 4.1. Full Demand Points Allocation

**Figure 4:**
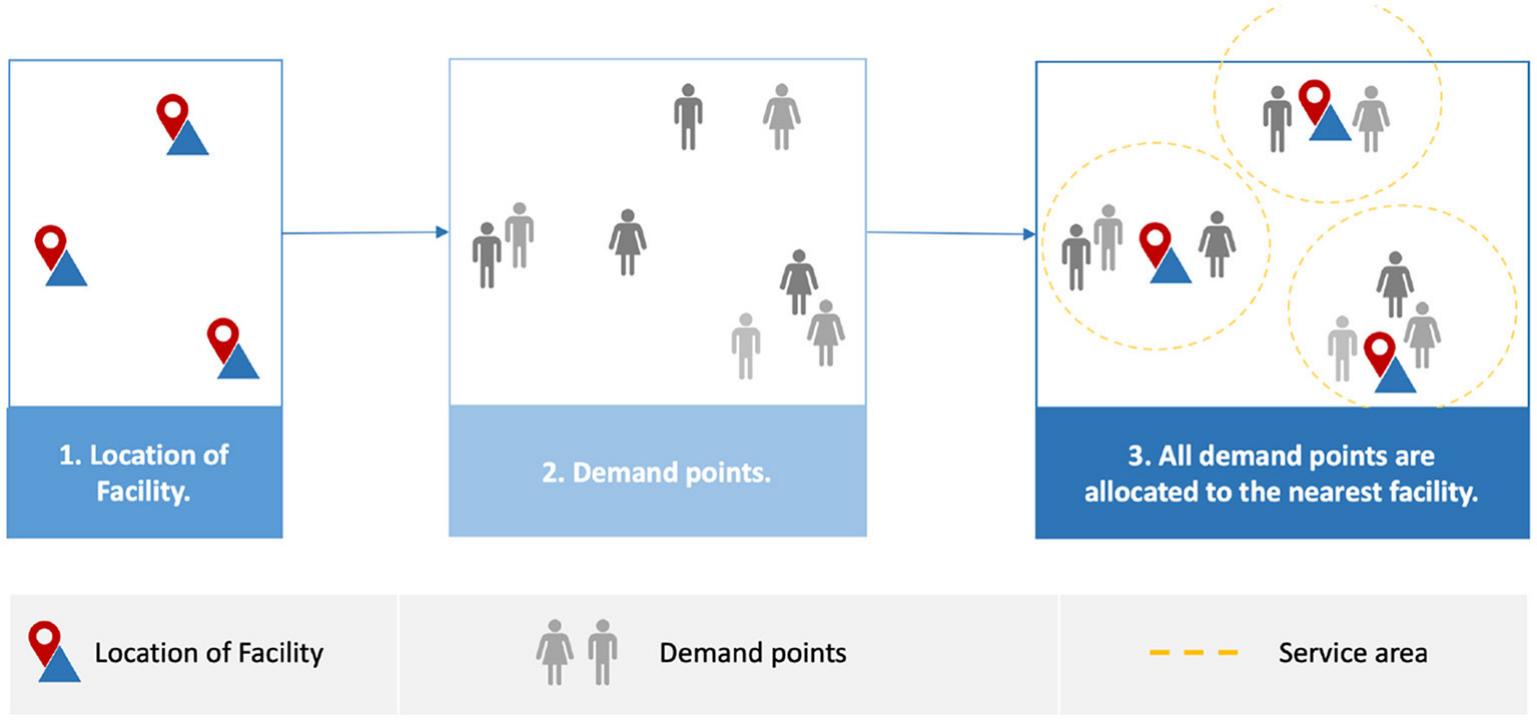
Full demand points allocation.

Full demand points allocation presents the solutions for locating the facilities regardless of the type of demand points. In other words, it provides equal access to PODs for all populations, as shown in Figure 4. Risanger et al. (23) suggested a model to select testing sites for COVID-19 using pharmacies to overcome the gap in coverage in the US in terms of the number of individuals who wish to perform testing at their nearest site and reduce the distance required to travel. They adopted an optimization model (24), which was used to optimize pharmacy-based distribution of antiviral drugs during the H1N1 pandemic. The objective is to maximize the number of people probably who can travel to limited testing sites (pharmacies). The optimization model is based on mixed-integer linear programming (MILP), they used mathematical optimization such as JuMP or CPLEX to solve it (25, 26), and implementation of solution is freely available via (25). The model used geographic region zip code and data with the population willing to travel as the input (24). The zip codes (mail delivery address) were converted to ZIP Code Tabulation Areas (ZCTAs). ZCTAs are used by the US Census Bureau to help obtain such information as counts, centroid coordinates, and latitudes and longitudes for each ZCTA. The pharmacy datasets were obtained from InfoGroup, Amazon Web Services, and Esri's national COVID-19 database. The results estimated that 94% of the population could access pharmacies, with coverage exceeding 80% and 90% for 47 and 12 states, respectively. However, the capacity of pharmacies is not considered, which may make it difficult to manage matching testing capacity with the testing demand.

In another study, Deng et al. (27) aimed to minimize the number of emergency medical service (EMS) facilities that need to be added to current hospital networks to cover 90% of the population within 15 min of travel time. The population and facility data were obtained from LandScan (28), the Health Bureau of Sichuan Province and the Health Bureau of Chengdu (not available to the public). The proposed solution used Nearest-Neighbor to find the nearest EMS using geographical information systems (GIS), with the shortest one used. To solve the location set covering formulation, a GA was then used. The results demonstrated that 55 of new EMS facilities were able to cover 90% of the population within 15 min. It was also found that to access an EMS facility, the weighted median shortest travel time were reduced by 14.57%. However, in some cases the road network may be affected by traffic jams, leading to an increase in access time.

Devi et al. (29) suggested a model based on MILP for temporary testing laboratories, aiming to minimize the total cost and the travel time from demand points to the laboratories. The overall cost includes fixed, operating, traveling and capacity underutilization costs. They applied the model to Holmberg et al. (30) and found underutilization of capacity. Based on that, they added a third decision variable to capture the capacity needs. The model was applied to a case study in Maharashtra, India, and the data are included in the paper. The results demonstrated that when there are 27 temporary testing laboratories, the total cost for the first objective is 6.08E + 11, while the second objective achieves 30.681 min. However, it would prefer to use ward-village level data to ensure that the centers area located near people.

### 4.2. Partial Demand Points Allocation

**Figure 5:**
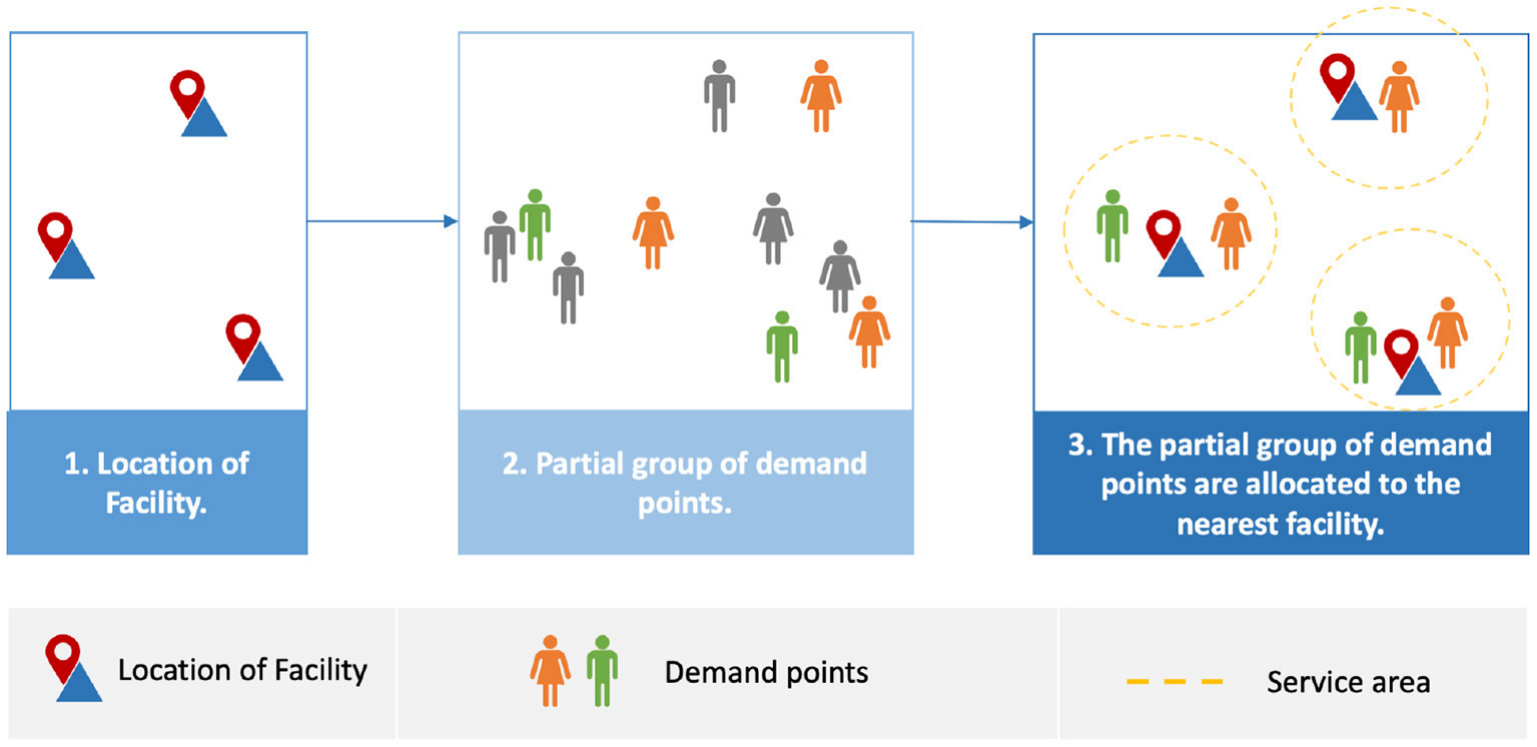
Partial demand points allocation.

**Figure 6:**
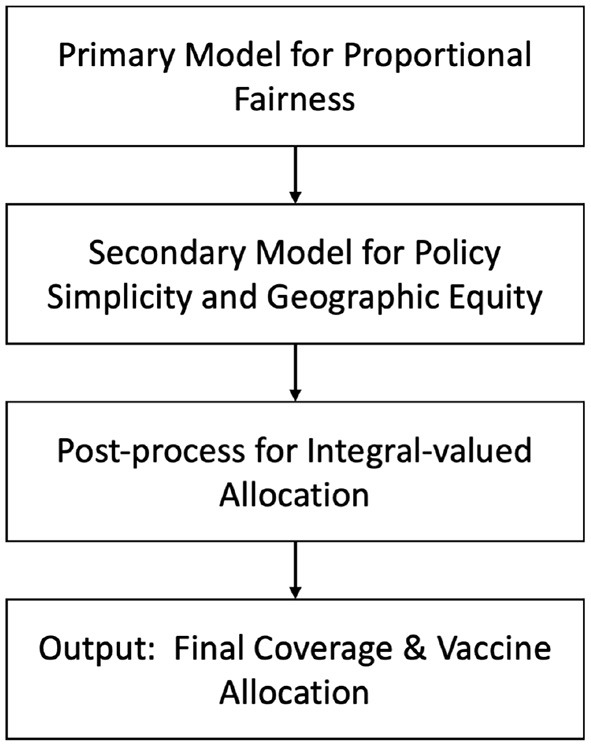
Model for allocating vaccines of multiple types to priority groups at multiple locations (31).

Partial demand points allocation considers a partial group of the population assigned to PODs, as shown in Figure 5, for example older people at high risk. Huang et al. (31) proposed a model for allocating four different types of vaccine to priority groups via PODs distribution in Texas in the US. There were five priority groups: ages 0–3, ages 4–24, high risk people aged 25–64, infant caregivers, and pregnant women. The model has three steps, as shown in Figure 6 (31), which are the primary optimization model, secondary optimization model, and post-process. The primary optimization model aims to produce an optimal coverage rate to achieve equal access for each location-priority group pair. The secondary optimization model then reduces the type of vaccine for each priority group and ensures geographic equity by providing similar proportions of vaccine allocation among regions. The final step in the model is the post-processing to obtain integer values for vaccine dose allocations; both primary and secondary optimization model ignores the integrality requirements. The model used priority group data (32) and healthcare locations where the vaccine was available using a tool designed for Texas (33). The results showed that with a small, reserved amount of vaccine (6.8%), the model satisfied the requirement of equal access, with 61.1% coverage of the priority population. However, the number of doses required to achieve immunity may reduce the chance of ensuring fair access for all communities.

Gao et al. (34) proposed two models for the temporary emergency center location-allocation such as M1 and M2. M1 aims to minimize travel time, whereas M2 aims to minimize the rate of mortality risk. Then, GA and modified fuzzy c-means (MFCM) (GA-MFCM) were developed. MFCM was modified to introduce the initial value for mortality risk. Whereas, GA was used to optimize the center of each cluster, and MFCM to group the data. They applied the proposed model in the form of a real dataset from Portland (America) post-earthquake emergencies (34). The results show the M1 model achieves less travel time than the M2 model, and M2 minimizes the total rate of mortality risk for patients. However, the injury severity level at the threshold of death is stated as a deterministic value.

In another study, Hudgeons (35) compared the use of the opened PODs only against using the Home Health Agency (HHA) as closed PODs and examined the impact on maximizing throughput (people's access to PODs) and minimizing resource allocation (staff). The suggested solution consists of replicating the process of services in PODs without HHA and identifying the necessary HHA nurse process. In replicating PODs, RealOpt simulation modeling software was used to show the patient's state traveling to, entering and leaving the PODs. The process of the HHA nurse was then defined as in-home and in-transit activities. For in-home activities, Monte-Carlo simulation was used to estimate the nursing processes required from entering to leaving the patient's home. For the transit modeling, traveling salesman heuristics were used to find the shortest path from the current location to the following location. Data from the nursing faculty at the University of Arkansas (not available to the public), the zip code for the HHA, and the squared area of each zip code (36) were used. The results showed that including the HHA as closed PODs increased throughput by 2.4% and required 16 fewer members of staff. Moreover, the solution targets the vulnerable group but is not based on a real-life case as such it does not specify who was involved and where they were located.

Memari et al. (37) suggested a solution to allocate injured people to temporary emergency stations, with four priorities group from high to low priority. They proposed a new fuzzy dynamic location-allocation with multi-medical servers M/M/c queue model. The model has bi-objectives to minimize the cost to construct temporary emergency stations and death rate and minimize the travel time and queue's response time. Furthermore, a fuzzy number is used to tackle treatment demand, travel time, and arrival treatment rate uncertainty. They used the augmented ε-constraint method to validate the model and a non-dominated sorting genetic algorithm (NSGA-II) to solve the problem; they applied the model in synthetic data and real case from Iran, Tehran (no information about the data). The results demonstrated that the model was able to achieve a good result to find the solutions. However, in some cases the road network may be destroyed due to disaster, leading to need for vehicle routing.

Li et al. (38) suggested a model that could achieve three objectives for locating vaccine sites to provide preventive vaccination to a specific group, in this case children. The three objectives were to minimize average travel distance to demand points, maximize the number of fully open vaccine stations, and minimize total costs, such as the fixed cost of opening the vaccine station or the cost of medical staff. A mixed-integer nonlinear programming (MINLP) model was used, which was divided into two stages to reduce the computational burden. Firstly, minimizing average travel distance is considered the main objective, and this objective and other objectives are solved independently using MILP; secondly, the other two objectives are considered constraints. The model was applied to Nanshan CDC in Shenzhen, China; the data for address and areas of vaccination stations are obtained from CDC. Vaccination stations, demand points and other data are available in (39). The results demonstrated that opening 50 vaccine sites involves a trade-off between three objectives, which helps CDC's decision-making. However, moving the demand point from one location to another may involve the need to re-assign people to a new and closer vaccine station.

Emu et al. (40) suggested the priority in conjunction with distance-based vaccine distribution model (PD-VDM) based on constraint satisfaction programming (CSP) to optimize the vaccine distribution, aiming to maximize distribution for priority groups. In this case, they considered age and minimized average travel distance for individuals to get vaccinated. The PD-VDM model is initialized by using k-medoid to select the number of distribution centers (DCs) and then the priority groups are assigned by minimizing the average distance. They performed two experiments based on the randomly generated data and real data obtained from Chennai, India. Additionally, they compared their model with other models using different factors, such as none or one of the distance and priority factors. They found that, their proposed model (using randomly generated datasets) was able to vaccinate the highest priority groups at 100% while reducing the average travel distance by more than 40%. However, in the real case, the model could vaccinate less than 90% of the three highest priority groups while reducing the average travel distance by more than 70%, which made the proposed model better than others. However, number of doses required to achieve immunity may reduce the chance of fair access for all communities and other groups needs.

Table 5 summarizes current work on location-allocation for PODs during public health emergencies in terms of reference, methodology, objective function, constraints, decision variables, type of the location-allocation model, datasets, and case study region (when applicable). The methodology is the solution for the proposed model. The objective function of the numerical value that could be maximized or minimized (42). Constraints are defined as the variable's possible values, while decision variables are the set of values that need to be defined to solve the problem (42). The location-allocation model is as described in Section 2.2. Datasets refer to the data used in the proposed model, while case study refers to whether the model is applied to a specific location, such as a particular city.

**Table 5:** Summary of location-allocation solutions for PODs during public health emergencies.

**Reference**	**Methodology**	**Objective function**	**Constraints**	**Decision Variables**	**Location- allocation model**	**Case Study**	**Datasets**
Huang et al. (31)	Optimization model	Maximize fair access for different priority groups	Discretionary doses	Regions for health service, vaccine types and priority groups	Coverage- based	Texas, US	Priority groups (32) healthcare places for vaccine (33).
Gao et al. (34)	GA and MFCM	Two-objectives: Minimize travel time Minimize the rate of mortality risk	Constraints: (2) and at least one patient assign to facility	*Y*_*ij*_	Median- based	Portland, US	Population and people geographical coordinates locations (34).
Hudgeons (35)	RealOpt simulation modeling, Monte-Carlo simulation and traveling salesman heuristics	Maximize throughput and minimize resource allocation	Maximum waiting time, required utilization per PODs, and number of workers (41)	Number of workers in each POD (41)	Coverage- based	-	Data from nursing faculty (not available to the public), zip code for HHA, and the squared area of each zip code (36).
Memari et al. (37)	NSGA-II, M/M/c	Bi-objectives: minimize the cost to construct emergency stations and death rate and to minimize the travel time and queue's response time	Total patient demands in a temporary station at time t, number candidate locations, patient allocates to the nearest and only one site, number of medical servers, waiting time, idle probabilities, cost of constructing a temporary station	A patient assigns to a temporary station at time t, a selected temporary station at time t, list of selected temporary stations at time t, number of temporary stations are selected from candidate locations, number of medical servers at time t in a temporary station	Coverage- based	Tehran, Iran	Simulated data source (37).
Risanger et al. (23)	Facility location optimization model	Maximize coverage for people want to perform the test	Constraints: (2), (3), (5), (6), (7) Population willing to travel (24).
Deng et al. (27)	Nearest- Neighbor and GA	Minimize number of new facilities	Percentage coverage	Candidate locations and the demand points are covered by candidate locations within 15 min	Coverage- based	Chengdu, China	Population data LandScan (28). Road network from National Geomatics Center of China. Supply side such as healthcare facilities from Health Bureau of Sichuan Province and the Health Bureau of Chengdu.
Li et al. (38)	MINLP model	Multi-objectives: minimize average travel distance for demand points, maximize the	open facility, fixed cost of opining facility and labor	Demands group, medical staff, fully opening facility, opening facility, workdays of	Median- based	Shenzhen, China	The address and areas of vaccination stations are obtained from CDC. Vaccination stations, demand points and
		Number of fully open vaccine stations, and minimize the total costs	cost	opening facility and order amount for each facility	Median- based	Shenzhen, China	other data are available in (39).
Emu et al. (40)	K-medoid and CSP	Maximize vaccine distribution for priority groups and minimize the average travel distance	One employee vaccinates one person, each person receives one vaccine from a single employee and the availability of the vaccine	The DC allocates an employee for the person to be vaccinated	Median- based and coverage- based	Chennai, India	Hospital locations distance from google map and age distribution during census 2011 (40).
Devi et al. (29)	MILP model	Bi-objectives: Minimize the cost and the travel time	Constraints: (2), (4), (5), fairness of access, number of facilities	*X*_*j*_, *Y*_*ij*_ and the capacity of facility	Coverage- based	Maharashtra, India	Holmberg et al. (30) and real data (29).

Table 6 presents a comparison of current work on location-allocation for PODs based on their advantages and limitations during public health emergencies. Table 7 compares studies based on a range of key features: locating facilities, identifying number of facilities, the number of new facilities, type of facility, density, and full or partial demand points allocation. In terms of locating facilities, the physical locations for PODs are selected, while the identifying number of facilities defines the number of PODs needed to accomplish the desired objective. Meanwhile, the number of new facilities identifies the number of new PODs needed in addition to the current set of facilities to accomplish the desired objective. The type of facility defines the place allocated to provide the service, such as a pharmacy. Finally, in terms of density, it is stated if the proposed solution covers different population densities.

**Table 6:** A comparison of location-allocation solutions for PODs during public health emergencies based on their advantages and limitations.

**Reference**	**Advantages**	**Limitations**
Huang et al. (31)	- Allocating the different types of vaccine (four types) for priority groups via PODs distribution in Texas, US.	- The number of doses required to achieve immunity may reduce the chance of fair access for all communities.
Gao et al. (34)	- Two objectives are considered as M1 and M2: M1 aims to minimize travel time, while M2 aims to minimize the rate of mortality risk.	- The injury severity level at the threshold of death is given as a deterministic value.
Hudgeons (35)	- Using closed PODs instead of only using opened PODs. - Including closed PODs with opened PODs increases throughput by 2.4% and decreased staff shortage by 16 persons.	- The solution targets the vulnerable group but is not based on a real-life case as such it does not specify who was involved and where they were located.
Memari et al. (37)	- Tackling treatment demand, travel time, and arrival treatment rate uncertainty. Risanger et al. (23)	- Selecting testing sites for COVID-19 using pharmacies to overcome gaps in coverage in the US. - 94% of the population could access pharmacies.	- The capacity of pharmacies is not considered, which may make it difficult to manage matching testing capacity with the testing demand.
Deng et al. (27)	- Minimizing the number of EMS facilities that need to be added to current hospital networks. - 55 of new EMS facilities are able to cover 90% of the population within 15 min of travel time.	- In some cases the road network may be affected by traffic jams, leading to an increase in access time.
Li et al. (38)	- Locating vaccine sites whilst minimizing average travel distance for demand points, maximizing the number of fully open vaccine stations, and minimizing total costs. - Opening 50 vaccine sites makes a trade-off among three objectives, which helps CDC decision-making.	- Moving the demand point from one location to another may involve the need to re-assign people to a new and closer vaccine station.
Emu et al. (40)	- Maximizing vaccine distribution for priority groups and minimizing the average travel distance.	- The number of doses required to achieve immunity may reduce the chance of fair access for all communities and other groups needs.
Devi et al. (29)	- Providing solution for temporary testing laboratories with aiming to minimize the cost and the travel time from demand points to the laboratories.	- It would prefer to use ward-village level data to ensure that the centers area located near people.

**Table 7:** A comparison of location-allocation solutions for PODs during public health emergencies based on their key features.

**Reference**	**Locating facilities**	**Identifying number of facilities**	**Number of new facilities**	**Type of a facility**	**Density**	**Full demand points allocation**	**Partial demand points allocations**
Huang et al. (31)	-	-	-	-	-	-	✓
Gao et al. (34)	✓	-	-	-	-	-	✓
Hudgeons (35)	-	-	-	HHA	-	-	✓
Memari et al. (37)	✓	-	-	-	-	-	✓
Risanger et al. (23)	✓	-	-	Pharmacy	✓	✓	-
Deng et al. (27)	✓	-	✓	-	-	✓	-
Li et al. (38)	✓	✓	-	-	-	-	✓
Emu et al. (40)	✓	✓	-	-	-	-	✓
Devi et al. (29)	✓	-	-	-	-	✓	-

## 5. Challenges and Future Research Directions

Section 4 presented different solutions currently proposed for the location-allocation of PODs during public health emergencies. However, certain issues and limitations remain that must be addressed in future studies. This section discusses these challenges and suggests future research directions for PODs location-allocation during public health emergencies.

- **Analysis of demand points**
  Demand for services in PODs will vary from country to country and from one kind of emergency event to another. For example, the COVID-19 vaccine is not appropriate for children, which may change the allocation decision. Existing solutions either assume that specific groups (38) are involved, or select them based on priorities in a specific type of emergency event (31).
  Thus, any analysis of the desired group (such as a priority or a vulnerable group) is very challenging and needs more attention due to the varied types of emergency (such as a pandemic or tsunami) and the needs of different groups of people (for example the elderly, children, or pregnant women). More importantly, the point or location of demands needs to be considered, along with whether it is easy to access the PODs or a new POD needs to be set up.
- **Covering different countries and population**
  Population size and density differ from one country to another; current solutions focus either on China (27, 38) or the US (23, 31). A few cases from other countries such as Iran and India (37, 40). A wider variety of solutions is needed to cover populations of different sizes and densities in other countries and cities in addition to China or the US. Doing so may help decision-makers to locate and utilize PODs according to the country's needs and to allocate them to populations of different sizes and densities.
- **Model constraints**
  PODs are locations for dispensing medical services, such as vaccines or drugs, to a large number of people while meeting a specific time constraint (4). As shown in Table 5, PODs location-allocation problem considers variety constraints such as budget or number of the fully open facility.
  Therefore, PODs location-allocation problem needs to consider other types of constraints related to PODs location-allocation problems, such as solving the problem with the required timeframe to ensure the coverage of either entire population or partial population. Also, the partial group such as vulnerable or priority groups can be considered a constraint for the model.
- **Evaluation and comparative studies**
  To address the location-allocation problem, Gwalani et al. (22) evaluated and compared four heuristic algorithms to resolve the p-median problem as applied to different terminologies, namely objective function value, time, and stability. However, there is a dearth of comparative studies evaluating methods utilizing several criteria, involving determining which methods are most applicable to maximize population coverage for populations of different sizes and densities. This will be a very interesting research direction for researchers seeking to provide data to evaluate and compare models and methods efficiently.

## 6. Conclusion

Public health emergencies such as disease outbreaks need PODs to dispense medical services such as vaccines to a large number of people within a specific period of time. PODs need to be situated in an optimal location and have demand points assigned to them simultaneously; this is known as the location-allocation problem. PODs may need to be selected to serve the entire population (full allocation) or different priority or needs groups (partial allocation). This paper presents a survey of PODs location-allocation models during public health emergencies and provides a taxonomy according to full and partial demand points allocation, and discusses a range of studies that have employed this taxonomy, comparing the advantages, limitations and key features of each one.

The survey concluded that in order to improve PODs location-allocation models during public health emergencies, there is a need to develop various techniques to analyze and define the demand of partial groups and provide the desired coverage to those demand points. Efforts to propose models to cover the needs of different countries, including variation in population size and density, are urgently needed. Furthermore, model constraints, such as time or priority groups need to be considered in the solution. Moreover, additional comparative studies are required to clarify which methods or models are adequate based on predefined criteria. Finally, this work has discussed the current challenges associated with existing techniques and recommended future research areas in PODs location-allocation models.

## Author Contributions

NA, RA, and SA: conceptualization and methodology. NA: formal analysis, investigation, writing—original draft preparation, and visualization. NA, RA, SA, AA, OB, and KF: resources, writing—review and editing, and funding acquisition. RA and SA: supervision. SA: project administration. All authors have read and agreed to the published version of the manuscript.

## Funding

The authors extend their appreciation to the Deputyship for Research & Innovation, Ministry of Education in Saudi Arabia for funding this research work through the project number IFPRC-055-612-2020 and King Abdulaziz University, DSR, Jeddah, Saudi Arabia.

## Conflict of Interest

The authors declare that the research was conducted in the absence of any commercial or financial relationships that could be construed as a potential conflict of interest.

## Publisher's Note

All claims expressed in this article are solely those of the authors and do not necessarily represent those of their affiliated organizations, or those of the publisher, the editors and the reviewers. Any product that may be evaluated in this article, or claim that may be made by its manufacturer, is not guaranteed or endorsed by the publisher.
